# Does minimed 780G^TM^ insulin pump system affect energy and nutrient intake?: long-term follow-up study

**DOI:** 10.1038/s41430-024-01422-y

**Published:** 2024-03-08

**Authors:** Yasemin Atik-Altinok, Yelda Mansuroglu, Gunay Demir, Hanife Gul Balki, Samim Ozen, Sukran Darcan, Damla Goksen

**Affiliations:** Division of Pediatric Endocrinology, Department of Pediatrics, Faculty of Medicine, Ege University, İzmir, Turkey

**Keywords:** Nutrition, Type 1 diabetes, Paediatrics

## Abstract

**Objective:**

We evaluate the energy and nutrient intake of children, adolescents, and young adults with type 1 diabetes (T1D) who started to use automated insulin delivery (AID) systems before the transition and during follow-up for 6 months in a real-world setting.

**Research design and methods:**

Twenty-nine people with T1D (PwD) who started to use MiniMed 780G^TM^ participated in the study. Participants’ 3-day food diaries and glycemic outcomes were analyzed at baseline and after (the 3^rd^ and 6^th^ month) switching to an advanced hybrid closed-loop system (a-HCL).

**Results:**

Mean carbohydrate, protein, and fat intake (energy %) at baseline were 49.1 ± 4.5, 17.8 ± 2.3, and 33.0 ± 3.9, respectively, and there were no statistically significant differences during the follow-up period. However, low fiber (<14 g/1000 kcal) and high saturated fat (>10 energy %) intake in PwD, both baseline and follow-up period. The median auto-correction bolus ratio was 14.0 (9.5)% at auto mode after 14 days, 18.0 (11.0)% at the 3^rd^ month, and 19.0 (7.5)% at the 6^th^ month (*p* < 0.05). A negative correlation was present between auto-correction boluses with TIR in both the 3^rd^ (r:-0.747, *p* < 0.01) and 6^th^ month (r:-0.395, *p* < 0.05). A negative correlation was present between auto-correction boluses with TIR in both the 3^rd^ (r:-0.747, *p* < 0.01) and 6^th^ month (r:-0.395, *p* < 0.05).

**Conclusions:**

a-HCLS systems offer better glycemic control. Using the Minimed 780 G^TM^ insulin pump system didn’t change the energy and nutrient intake of PwD. This real-world follow-up study suggests that children, adolescents, and young adults with T1D consume saturated fat above and fiber intake lower than recommendations independent of the use of a-HCLS.

**Clinical trials registration number:**

NCT05666596.

## Introduction

The use of AID systems in individuals with T1D should be strongly considered by clinicians because these systems improve glycemic control and quality of life [1]. AID systems are recommended for all youth with patients with diabetes by the International Society of Pediatric and Adolescent Diabetes (ISPAD) Clinical Practice Consensus Guidelines 2022 [2]. AID systems utilize an algorithm that continuously adjusts insulin delivery in response to real-time glucose monitoring system data, residual insulin action, and other inputs, such as meal intake and exercise announcements. However, despite significant advances in controller algorithms in providing closed-loop insulin delivery between meals, users must still manually announce carbohydrate intake to achieve adequate postprandial insulin coverage [1].

Nutrition management of T1D is the cornerstone for optimal glycemic control, and dietary recommendations are based on healthy eating principles suitable for all children and adolescents with T1D. Although the optimal macronutrient distribution varies depending on an individualized assessment of the PwD, ISPAD advises as a guide carbohydrate should approximate 40–50% of energy, fat <35% of energy (saturated fat <10%), and protein 15%–25% of energy. These recommendations target healthy eating principles, glycemic management, reducing cardiovascular risk factors, and maintaining psychosocial well-being [3].

The AID system has been shown to be safe and to significantly improve glycemic control compared to baseline or control treatment groups in clinical trials with children, adolescents, and adults. Real-world data is now available, shedding light on true AID acceptance and performance [4–8].

Although the importance of accurate carbohydrate counting was emphasized in the training given before the transition to a-HCLS, some of the young people stated that they had unrealistic expectations that this would not be so important due to the auto-correct feature of the system before switching to the pump, most people stated that they could eat junk foods and high-fat foods as much as they wanted with this smart pump, without hyper or hypoglycemia. When we realized these expectations of PwD and some parents/caregivers, we hypothesized that AID might affect energy and nutrient intake in ways that increase fat intake. Based on this hypothesis, we decided to evaluate the energy and nutrient intake with 3-day food diaries at the baseline, 3^rd^, and 6^th^ month, which we predict will reflect the food consumption of individuals with diabetes.

There are several studies assessing the impact of CGM and/or pump use on nutrient intake [9–11]. However, although studies conducted with AID systems show improvements in glycemic outcomes regardless of gender, age group, duration of diabetes, previous insulin delivery method, or baseline HbA1c levels, they don’t contain data on the effect on food intake. We aimed to determine whether there is a change in the macronutrient and fiber intakes of children and adolescents with type 1 diabetes who started using the AID system in order to see real-life data.

## Materials and methods

### Study design and participants

This 6-month follow-up study was conducted with twenty-nine children, adolescents, and young adults with T1D between November 2021 and May 2022. In our clinic, in November-December 2021, thirty-eight PwD switched to using a-HCLS. The data of twenty-nine cases with the Minimed 780G insulin pump system that was in adherence to the study protocol during the follow-up period were included in the analysis. Exclusion criteria included co-morbidities (celiac disease, cystic fibrosis, etc.) affecting food consumption and nutrient intake.

All participants and caregivers participated in a training session to start the system in manual mode and understand its functions. The auto mode was initiated after 3 days for sensor-augmented pump therapy (Minimed 640G^TM^, Medtronic, Northridge, CA USA) users and 10 days for multiple daily injections (MDI) users. During this period, and the 3^rd^ and 6^th^ month of the a-HCLS initiation, the participants were asked to write a 3-day food diary.

### Anthropometric evaluation

Height was measured to the nearest millimeter using a Seca 264^®^ stadiometer. Weight was measured unclothed using an electronic scale to the nearest 100 g (Desis Model KW^®^). Body mass index (BMI) was calculated by the weight (kg)/height (m²) equation. Standard deviation scores (SDS) for weight, height, and BMI were calculated according to age and gender using reference values for Turkish children and adolescents. For children and adolescents, BMI-SDS ≥ -1 - <+1, and for young adults, a BMI of 18.5–24.9 kg/m² is considered normal weight and calculated by an automated program [12–14].

### Food diary

Dietary intake was evaluated for each participant by filling out 3-day food diaries, including three consecutive days (two weekdays and one weekend day). Parents of children with T1D, adolescents, and young adults with T1D were given oral sessions and written instructions on the method for weighing and recording food by the diabetes team dietician. All food and beverages (including dressings) consumed were recorded by weighing for 3 days each at baseline of switching to Minimed 780G^TM^, 3^rd^ and 6^th^ month (9 days for each participant). Food diaries were controlled by 2 dietitians, verified for consistency and accuracy, and asked for supplementary information if needed. The analysis included dietary records of 1076 snacks and meals (750 meals, 326 snacks). Total energy intake (kcal), carbohydrate (energy %), protein (energy %), fat (energy %), saturated fatty acids (energy %), dietary cholesterol (mg), and dietary fiber (g/1000 kcal) intake were calculated using the Ebispro for Windows; Turkish Version (BeBiS 8.2) (Stuttgart, Germany).

### Glycemic parameters

HbA1c was measured by turbidimetric inhibition immunoassay (Roche Cobas c513 analyzer using the Tina quant® HbA1c Gen. 3 assay, Germany). MiniMed 780G^TM^ data uploaded to CareLink^TM^ personal software during the follow-up by individuals who provided consent for their data to be aggregated were analyzed (CareLink; https://carelink.medtronic.eu). TIR (70–180 mg/dl), TBR (<70 mg/dl), time above range (TAR: >180 mg/dl), coefficient of variation (CV), glucose management indicator (GMI), sensor wear, time spent in a closed loop, auto bolus % and sensor glucose values were evaluated. Active insulin time was 2,5 h, and the target blood glucose value was 100 mg/dl in all patients at the initiation of a-HCLS and changed when necessary.

### Insulin delivery

MDI patients were on intensive insulin therapy (glargine and aspart/glulisine/lispro), and pump patients were on Minimed 640G^TM^ insulin infusion pump. Participants’ carbohydrate counting accuracy and consistency were evaluated before initiating a-HCLS.

### Statistical analysis

Statistical analyses were conducted using Statistical Package for the Social Sciences version 25.0 (SPSS Inc., Chicago, IL, USA). The level of significance was defined as *p* < 0.05. Categorical variables were represented as counts and percentage values. Normal distribution was tested for quantitative variables. Continuous variables with normal or skewed distribution were presented as mean ± SD or median (IQR). Group differences were investigated using the independent *t* test for normally distributed data and the Mann–Whitney test for skewed data. Repeated value differences were analyzed using repeated-measures ANOVA for normally distributed data and the Friedman test for skewed data. Pairwise comparisons were tested using Bonferroni and Dunn’s test. Correlation analyses were used to explore relationships between carbohydrate, protein, fat, dietary fiber intake, and other constructs hypothesized to covary with macronutrient intakes such as TIR, TAR, TBR, CV and GMI in line with Cohen coefficient (0.10–0.29 as small, 0.30–0.49 as medium, and 0.50–1.0 as large size) In post-hoc power analysis, eta squared (η2) was calculated to determine the effect size in repeated measurements (0.01 as small, 0.06 as medium, and 0.14 as large effect) was used.

Our main hypothesis in this study is that there will increase in the quantity of dietary fat intake by PwD when switching to the a-HCLS. Our main hypothesis in this study is that there will be an increase in the amount of dietary fat consumed by participants when switching to the a-HCLS. In repeated measurements, the η2 value was calculated as 0.046, and when converted to Cohen’s *f* value, it was obtained as *f* = 0.2125. Accordingly, in repeated measurements (3 repetitions), with an effect size of *f* = 0.2195, α = 0.05 type 1 error, the post-hoc power value for 1 group and 3 repeated measurements was calculated as 0.7146.

## Results

The mean age of 29 PwD was 12.7 ± 4.3 years (min 5- max 22 years) (*n* = 14; 48.3% female), the median diabetes duration was 2.2 (4.1) years, and the mean HbA1c level was 6.9 ± 1.2%. Before switching to the AID system, 20% of the participants were on MDI therapy (≥4 daily injections), while 80% were on sensor-augmented pump therapy (Minimed 640G^TM^). There were no significant differences in age, diabetes duration, HbA1c levels, insulin requirements, and the number of meals/snacks per day between MDI users and pump users at the beginning and follow-up (Table 1). All participants had normal BMI values according to their age and gender and did not change throughout the follow-up period [*p* = 0.25 for <18 years of age (*n* = 22), and *p* = 0.087 for>18 years of age (*n* = 3)].

**Table 1:** Baseline characteristics of participants according to treatment groups.

	All (*n* = 29)	MDI (*n* = 6)	CSII (*n* = 23)	*p* value
Age (years)^a^	12.5 ± 4.8	11.8 ± 5.9	12.5 ± 4.5	0.914^c^
Diabetes duration (years)^b^	2.2 (4.1)	1.6 (4.9)	2.6 (5.6)	0.333^d^
HbA_**1**_c (%)[mmol/mol]^a^	6.9 ± 1.2 [51.9 ± 13.1]	7.4 ± 1.6 [56.8 ± 17.5]	6.8 ± 1.3 [50.8 ± 14.2]	0.957^c^
Insulin (U/kg/d)^a^	0.7 ± 0.3	0.7 ± 0.4	0.7 ± 0.2	0.987^c^
Number of meals per day^b^	3.0 (0.0)	3.0 (0.0)	3.0 (0.0)	0.220^d^
Number of snacks per day^b^	1.3 (1.8)	0.6 (2.7)	1.3 (1.7)	0.684^d^

*p* values refer to the significance of the difference between MDI users and pump therapy users.

^a^Data are mean ± standard deviation.

^b^Data are median (Interquartile range).

^c^Independent sample *t* test.

^d^Mann–Whitney U test.

The energy and nutrient intake of participants during the follow-up period are presented in Table 2. The participants’ carbohydrate, fat, and protein intakes met the recommended levels of national and international guidelines at baseline and during follow-up, and there were no statistically significant differences during the follow-up period [3, 15]. Although not statistically significant, fat intake increased during the follow-up period. In addition, saturated fat intake was above, and fiber intake was lower than the recommendations for both baseline and follow-up [3, 15]. The mean number of meals and snacks did not change during the follow-up. When the relationships between glycemic metrics and macronutrient intake were examined, a medium-sized positive correlation was observed between dietary protein intake and HbA1c before switching to a-HCLS (r:0.412,*p* < 0.05). While there was no relationship between macronutrient intake and glycemic metrics in the 3^rd^ month of follow-up, a medium-size negative correlation was detected between carbohydrate intake and TBR in the 6^th^ month of the follow-up period (r:-0.403, *p* < 0.05). There was a negative large-size correlation between dietary carbohydrate and fat intake, at baseline and throughout follow-up period, consistently (Table 3).

**Table 2:** Energy and nutrient intake of participants during the follow-up period.

	ISPAD recommendations	Beginning (0)	3^rd^ month (3)	6^th^ month (6)	*p* value	Pairwise comparisions	Effect size (η2)
Energy (kcal/d)^a^	Varies according to age, gender and physical activity level	1520 (362)	1479 (421)	1519 (570)	0.891^c^	NS	
Protein (energy %)^b^	15–25	17.8 ± 2.3	16.4 ± 2.5	17.6 ± 3.2	**0.041**^d^	0–3: p = **0.031**^e^ 3–6: p = **0.02**^e^	0.108
Carbohydrate (energy %)^b^	40–50	49.1 ± 4.5	48.9 ± 7.1	47.5 ± 6.6	0.416^d^	NS	0.031
Fat (energy %)^b^	35–40	33.0 ± 3.9	34.8 ± 6.5	35.0 ± 5.9	0.264^d^	NS	0.046
SFA (energy %)^a^	<10	12.4 (5.2)	11.6 (4.9)	12.8 (4.1)	0.439^d^	NS	0.065
Dietary cholesterol (mg)^b^	<300	319.2 ± 169.7	283.6 ± 137.7	288.5 ± 134.7	0.223^c^	NS	0.006
Dietary fiber (g/1000 kcal)^a^	14	11.4 (3.9)	11.2 (2.6)	11.4 (2.9)	0.995^c^	NS	0.002
Number of meals per day^a^	No recommendation	3.0 (0.0)	3.0 (0.0)	3.0 (0.2)	0.368^c^	NS	0.026
Number of snacks per day^a^	No recommendation	1.3 (1.8)	1.0 (1.4)	1.0 (1.3)	0.443^c^	NS	0.059

*SFA* saturated fatty acid, *NS* Non significant.

^a^Data presented as median (IQR).

^b^Data was presented as mean ± SD.

^c^Friedman’s test.

^d^Repeated-measures ANOVA.

^e^Paired sample *t* test.

bold values indicate statistical significance.

**Table 3:** Correlation between macronutrient intake and glycemic metrics.

	1	2	3	4	5	6	7	8	9	10
a: Correlation between macronutrition intake and glycemic metrics at the baseline										
1. Protein (energy %)	1									
2. Carbohydrate (energy %)^b^	−0.480^**^	1								
3. Fat (energy %)^b^	0.008	−0.859^**^	1							
4. Dietary fiber (g/1000 kcal)^a^	0.139	0.095	−0.134	1						
5.HbA1c (%)	0.412*	−0.328	0.118	−0.292	1					
6. TIR	−0.154	0.014	0.029	0.230	−0.109	1				
7. TAR	0.159	−0.053	0.015	−0.347	0.096	−0.907^**^	1			
8. TBR	0.126	0.056	−0.170	0.238	0.001	0.506	−0.396^*^	1		
9. CV	0.388	−0.199	0.018	0.125	0.384	−0.706^**^	0.382	0.640^**^	1	
10. GMI	−0.121	−0.031	0.135	0.899	−0.197	−0.489^**^	0.701^**^	−0.458^*^	−0.046	1
b: Correlation between macronutrition intake and glycemic metrics at the 3^rd^ month.										
1. Protein (energy %)	1									
2. Carbohydrate (energy %)^b^	−0.222	1								
3. Fat (energy %)^b^	−0.139	−0.896^**^	1							
4. Dietary fiber (g/1000 kcal)^a^	0.376*	0.340	−0.463^*^	1						
5.HbA1c (%)	0.110	0.312	−0.477	0.183	1					
6. TIR	0.186	−0.292	0.114	0.139	−0.514	1				
7. TAR	−0.214	0.340	−0.169	−0.156	0.589	−0.884^**^	1			
8. TBR	0.291	−0.114	0.007	0.249	0.146	0.047	−0.270	1		
9. CV	−0.142	−0.032	0.153	−0.146	0.497	−0.554^**^	0.349	0.201	1	
10. GMI	−0.356	0.321	−0.135	−0.154	0.328	0.676^**^	0.847^**^	−0.604^**^	0.168	1
c: Correlation between macronutrition intake and glycemic metrics at the 6^th^ month.										
1. Protein (energy %)	1									
2. Carbohydrate (energy %)^b^	−0.444^*^	1								
3. Fat (energy %)^b^	−0.057	−0.811^**^	1							
4. Dietary fiber (g/1000 kcal)^a^	0.119	0.423^*^	−0.504^**^	1						
5.HbA1c (%)	0.083	0.037	−0.146	−0.098	1					
6. TIR	−0.458^*^	−0.021	0.356	−0.115	−0.443^*^	1				
7. TAR	0.148	0.195	−0.366	0.192	0.491^*^	−0.819^**^	1			
8. TBR	0.440^*^	−0.403^*^	0.185	−0.184	−0.171	0.056	−0.496^**^	1		
9. CV	0.213	−0.181	−0.019	−0.054	0.252	−0.501^**^	0.087	0.409^*^	1	
10. GMI	−0.110	0.212	−0.296	0.186	0.453^*^	−0.564^**^	0.842^**^	−0.630^**^	−0.143	1

*TIR* time in range, *TAR* Time above range, *TBR* Time below range, *CV* Coefficient of variation, *GMI* Glucose management index.

^*^p < 0.05, ^**^p < 0.001.

According to the food diary, the amount of carbohydrates announced was consistent with the carbohydrate intake of the participants before they switched to auto mode. In contrast, the amount of carbohydrates announced to the pump was statistically higher than that recorded in the food diary on the 3^rd^ and 6^th^ months of follow-up (Table 4).

**Table 4:** Comparison of Daily Amounts of Carbohydrate Intake.

	Carbohydrate intake announced to pump (g/day)	Carbohydrate intake recorded in the food diary (g/day)	*p* value^a^
	Mean ± SD	Mean ± SD	
Baseline	119.2 ± 80.3	187.4 ± 45.0	0.361
3^rd^ month	220.9 ± 104.1	184.7 ± 44.2	**0.036**
6^th^ month	230.1 ± 112.7	184.3 ± 59.3	**0.011**

*SD* standard deviation.

^a^Paired sample *t* test.

bold values indicate statistical significance.

**Fig. 1:**
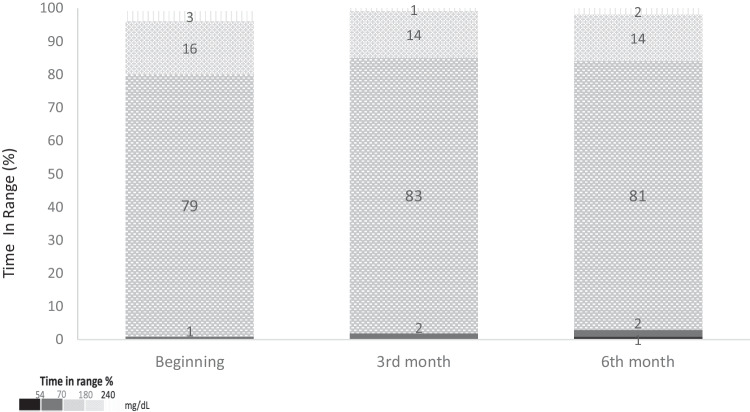
Histogram presentation of glycemic parameters. Histogram presentation of percentages of TIR, TAR, and TBR of all subjects by follow-up periods.

Median (IR) TIR increased from 79.0 (15.5)% to 81.0 (6.5)% (*p* < 0.05), and median (IR) GMI decreased from 6.6 (0.4) to 6.5 (0.3) *p* < 0.05 (Fig. 1). Basal and bolus insulin ratios were similar during follow-up.

The median auto-correction bolus ratio was 14.0(9.5)% at auto mode after 14 days, 18.0 (11.0) % at the 3^rd^ month, and 19.0 (7.5)% at the 6^th^ month (*p* < 0.05). There was a positive correlation between auto-correction boluses with TAR (r:0.775, *p* < 0.01), GMI (r:0.691, *p* < 0.01), mean blood glucose (r:0.527, *p* < 0.01), SD (r:0.491, *p* < 0.01) at 3^rd^ month and TAR (r: 0.440, *p* < 0.05) and GMI (r: 0.529, *p* < 0.01) on 6^th^ month. A negative correlation was present between auto-correction boluses with TIR in both the 3^rd^ (r:-0.747, *p* < 0.01) and 6^th^ month (r:-0.395, *p* < 0.05) (Table 5).

**Table 5:** Comparisons of glycemic outcomes at different time points.

	Manual mode (0)	Auto mode 3^rd^ month (3)	Auto mode 6^th^ month (6)	*p* value	Pairwise comparisons
	Median (IQR)	Median (IQR)	Median (IQR)		
GMI	6.6 (0.3)	6.4 (0.2)	6.5 (0.2)	**0.016**^a^	0–6: *p* = **0.043**
CV (%)	31.6 (4.1)	31.7 (3.1)	33.4 (3.2)	**0.039**^a^	NS
Mean blood glucose (mg/dL)	143.9 (14.2)	136.0 (12.5)	140.7 (12.3)	**0.035**^a^	0–3: *p* = **0.038**
SD (mg/dl)	43.9 (6.6)	41.9 (5.8)	42.2 (4.8)	0.127	NS
HbA1c^a^ (%) (mmol/mol)	6.9 (1.2) 54.2 (9.0)	6.7 (0.6) 57.5 (8.3)	6.7 (1.3) 51.1 (8.8)	0.575	NS
Bolus insulin (%TDI)	60.0 (17.5)	62.0 (8.5)	62.0 (11.0)	0.149	NS
Auto mode (%)	-	100.0 (3.0)	98.0 (5.0)	0.204^b^	NS
Sensor wear (%)	94.5 (4.5)	94.0 (5.0)	92.5 (8.0)	0.961^a^	NS

*IQR* interquartile range, *GMI* Glucose management index, *CV* Coefficient of variation, *SD* Standard deviation, *TDI* Total daily insulin.

^a^Wilcoxon signed rank test.

^b^Friedman test.

bold values indicate statistical significance.

## Discussion

This study aimed to evaluate the energy and nutrient intake of children, adolescents, and young adults with T1D switching to AID systems in real-life settings. To the authors’ knowledge, this is the first study to demonstrate the affect of using the AID system on energy and nutrient intake, which may reflect food preferences.

Nutrition management recommendations for children and adolescents with diabetes reflect guidelines for healthy eating developed for the general population. The optimal macronutrient distribution varies depending on the individualized assessment and metabolic priorities of the child and adolescent with T1D. However, the International Society of Pediatric and Adolescent Diabetes (ISPAD) gives the following thresholds as a guide: ‘carbohydrate intake should be 40–50% of total daily energy intake, fat intake no greater than 30–40% (saturated fat <10%), and protein intake 15–25%’ [3]. In our study, the participants’ carbohydrate, fat, and protein intakes met the recommended levels of national and international guidelines, both at baseline and during the follow-up [3, 15]. However, our findings about low fibre and high saturated fat intake in children with T1D, both baseline and follow-up period, supported previous studies [9–11, 16–19]. Dietary factors that raise VLDL and LDL cholesterol make prone to the formation of atherosclerosis in teenagers and young adults, and the amount of saturated fat in the diet is one of the main determinants of plasma LDL cholesterol level [20]. The American Heart Association advises children to consume a healthy diet that limits saturated fat and recommends replacement with polyunsaturated and monounsaturated fat to reduce cardiovascular disease (CVD) risk in later life [21]. These findings associated with participants’ high saturated fat intake have potential implications for clinical practice and nutritional education ingredients. To maintain a healthy intake, consumption of foods high in saturated fat should be limited, and children and adolescents with T1D should be supported in consuming legumes, fruits and vegetables, and whole grains containing fiber.

In our study group, with the AID system, TIR improved after switching, and this was maintained during the study period. TIR improvement was paralleled by a significant decrease in level 2 TAR without a significant increase in TBR. Similar results were reported by Silva et al. in a real-world evaluation of the performance of a-HCLS 4120 users from 8 countries (without demographic data), with the mean TIR increasing from 63.4 ± 14.3% to 75.5 ± 9.6% [6]. Piccini et al. reported 44 children and adolescents switching from MDI and CSII to a-HCLS with a follow-up period of 6 months, reporting a mean TIR increase from 69.3 ± 12.6% to 76.9 ± 8.7% [22]. Similar to previous studies, a statistically significant improvement in TIR and GMI were observed in our study group after switching to the AID system. The improvement of TIR was observed as 4% in the 3^rd^ month and 2% in the 6^th^ month. Unlike previous studies, the difference in improvement of TIR after switching to the AID system was low. The authors thought that the reason for this low level of improvement was related to the fact that the participants’ TIR levels already met international recommendations before switching to the AID system [23].

In line to previous studies, in our study group, the median percent of time in auto mode was 100.0 (3.0) at the 3^rd^ month and 98.0 (5.0) at the 6^th^ month [6, 8]. This can be explained by the high motivation of children, adolescents, and their families who switched to the AID system and by the fact that they can reach the diabetes team 24/7 in our clinic.

Similar to what Silva et al. [6] and Piccini et al. [22] reported in our findings, auto-correction boluses were approximately 20% (18% at 3^rd^ month, 19% at 6^th^ month). This negative correlation between TIR and auto boluses could be clarified by the fact that auto-correction intervenes when the user is inaccurate in counting carbohydrates at meals or when meal boluses are skipped/forgotten. As an unrealistic expectation, users expect from the system that there is no need to announce carbohydrates. These PwD are those who would benefit the most from targeted educational interventions during follow-up, and an “auto-correction bolus threshold” could be helpful in identifying and monitoring them.

It was observed that before switching to auto-mode, the mean carbohydrate quantity in the food diaries and announced to the a-HCLS were similar. In contrast, after switching to auto-mode, the carbohydrate announced to the pump was higher than the mean carbohydrate quantity in the food diaries. We speculate that this difference was due to the carbohydrate announced without eating to compensate hyperglycemia resulting from the forgotten or skipped carbohydrate. The negative correlation between dietary carbohydrate intake and TBR at the 6^th^ month, when the mean difference between the amount of carbohydrate announced to the a-HCLS and the quantity of carbohydrate intake from the diet was largest (-45.7 ± 90.9 g/day), may be a sign that tricking the system is causing deterioration of glycemic control. Therefore, it should be emphasized to children, adolescents, and caregivers that carbohydrate intake should be announced to the a-HCLS by the diabetes team. Users should be advised to avoid tricking the system.

The current analysis has strengths and limitations. The strength of the study is the prospective long-term follow-up (6 months) in a real-world setting and evaluation of energy and nutrient intake based on 3-day food diary records at baseline, 3^rd^ and 6^th^ months. The main limitation is the sample size. Additional evaluations with a larger sample should be performed to confirm these results. Another limitation of the study is that although it was conducted in real-life conditions, physical activity diaries were not collected. With this, participants reported that they continued their daily physical activity routines during the study period.

## Conclusion

Although the AID system offers flexibility around mealtimes and better glycemic control, the energy intake and macronutrient distribution of the diet of children, adolescents, and young adults with T1D didn’t change with the AID system. However, the food choices of PwD should be closely monitored by food diaries, and reduced saturated fat intake and increased fiber intake should be encouraged to minimize the risk of cardiovascular disease.

## Data Availability

Data generated analyzed during this study can be available from the corresponding author on reasonable request.
